# Clinical Implications of Bifurcation Angles in Left Main Bifurcation Intervention Using a Two-Stent Technique

**DOI:** 10.1155/2020/2475930

**Published:** 2020-07-11

**Authors:** You-Jeong Ki, Ji Hyun Jung, Jung-Kyu Han, Sukkeun Hong, Jang Hyun Cho, Hyeon-Cheol Gwon, Sung Yun Lee, Jay Young Rhew, Jei Keon Chae, In-Ho Chae, Han-Mo Yang, Kyung Woo Park, Hyun-Jae Kang, Bon-Kwon Koo, Hyo-Soo Kim

**Affiliations:** ^1^Cardiovascular Center, Seoul National University Hospital, Seoul 03080, Republic of Korea; ^2^Division of Cardiology, Department of Internal Medicine, Sejong General Hospital, Bucheon 14754, Republic of Korea; ^3^Department of Internal Medicine, St. Carollo Hospital, Sunchon 57931, Republic of Korea; ^4^Heart Vascular Stroke Institute, Samsung Medical Center, Sungkyunkwan University School of Medicine, Seoul 06351, Republic of Korea; ^5^Inje University Ilsan Paik Hospital, Goyang 10380, Republic of Korea; ^6^Division of Cardiology, Department of Internal Medicine, Presbyterian Medical Center, Jeonju 54987, Republic of Korea; ^7^Chonbuk National University Hospital, Jeonju 54907, Republic of Korea; ^8^Seoul National University Bundang Hospital, Sungnam 13620, Republic of Korea

## Abstract

**Objectives:**

The aim of this study was to assess the clinical impact of 3 bifurcation angles in left main (LM) bifurcation treated with the 2-stent technique.

**Background:**

Data are limited regarding the impact of bifurcation angles after LM percutaneous coronary intervention (PCI).

**Methods:**

Using patient-level 4 multicenter registries in Korea, 462 patients undergoing LM bifurcation PCI with the 2-stent technique were identified (181 crush, 167 T-stenting; 63% 1^st^ generation drug-eluting stent (DES), 37% 2^nd^ generation DES). Three bifurcation angles, between the LM and left anterior descending (LAD), the LM and left circumflex (LCX), and the LAD and LCX, were measured. The primary outcome was target lesion failure (TLF), a composite of cardiac death, myocardial infarction, and target lesion revascularization (TLR).

**Results:**

In patients treated with the crush technique, the best cutoff value (BCV) to predict TLF was 152° of the LM-LAD angle. In the crush group, a significantly higher TLF rate, mostly driven by TLR, was observed in the LM-LAD angle ≥152° group compared with the <152° group (35.7% vs. 14.6%; adjusted hazard ratio 3.476; 95% confidence interval 1.612–7.492). An LM-LAD angle ≥152° was an independent predictor of TLF. In the T-stenting, no bifurcation angle affected the clinical outcomes.

**Conclusions:**

In LM bifurcation PCI using the 2-stent technique, wide LM-LAD angle (≥152°) was associated with a greater risk of TLF in the crush, whereas none of the bifurcation angles affected T-stenting outcomes.

## 1. Introduction

Bifurcation disease remains a challenging lesion subset posing a higher risk of adverse events in the drug-eluting stent (DES) era [1, 2]. Although use of the provisional 1-stent technique has been widely recommended for percutaneous coronary intervention (PCI) of bifurcation lesions [3–6], the 2-stent technique is frequently necessary and justified [7], especially in left main (LM) bifurcation disease because of the importance of preserving the left circumflex (LCX) artery [8]. Among the potential factors affecting clinical outcomes of LM bifurcation PCI, bifurcation angle has drawn interventionists' interest. However, its impact has not yet been fully elucidated. Practically, one of the important factors in the stent strategy selection process is bifurcation angle: for example, T-stenting is considered appropriate for bifurcation with a near 90° angle between the main branch (MB) and side branch (SB) [3]. However, this practice is based on theoretical assumption without thorough validation using real-world data. Moreover, a few previous studies regarding bifurcation angle used heterogeneous definitions of the angle and reported controversial data [9–17]. Here, we sought to comprehensively assess the clinical impact of 3 different bifurcation angles (angles between the LM and the left anterior descending (LAD) artery, between the LM and the LCX, and between the LAD and the LCX) in patients undergoing LM bifurcation PCI using the 2-stent technique. Because visual estimation is widely adopted to assess bifurcation angles and determine a type of 2-stent technique in real-world practice, we used 2-dimensional quantitative coronary angiography (QCA) to measure bifurcation angles, results of which should be applied to daily practice.

## 2. Materials and Methods

### 2.1. Study Population

**Figure 1:**
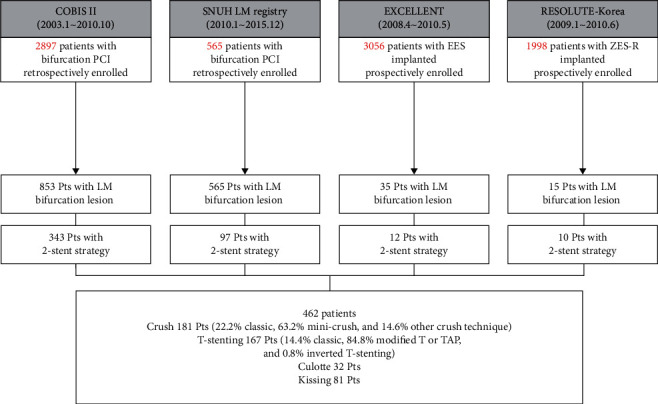
Study population from 4 multicenter registries. EES, everolimus-eluting stent(s); LM, left main; PCI, percutaneous coronary intervention; TAP, T and protrusion; ZES-R, zotarolimus-eluting resolute stent.

We analyzed patient-level pooled data from 4 multicenter registries in South Korea (Figure 1). The COBIS (COronary BIfurcation Stenting) II registry (NCT01642992) is a retrospective multicenter registry of individuals with coronary bifurcation lesions who underwent PCI with DES. Consecutive patients from 19 major coronary intervention centers in Korea were enrolled in this study between 2003 and 2010. The inclusion criteria were (1) age ≥18 years; (2) coronary bifurcation lesions treated with DES; and (3) a side branch or LCX reference diameter ≥2.3 mm and at least stentable with a 2.5 mm stent. The exclusion criteria were (1) protected LM disease (previous coronary artery bypass grafting in the LAD or LCX territory); (2) cardiogenic shock; and (3) history of cardiopulmonary resuscitation in the same hospitalization. The Seoul National University Hospital (SNUH) LM registry is a retrospective registry of patients undergoing PCI of bifurcation lesions at SNUH. From 2010 through 2015, a total of 565 patients were enrolled in this registry. The EXCELLENT (Efficacy of Xience/Promus Versus Cypher in rEducing Late Loss After stENTing) Registry (NCT00960648) and RESOLUTE-Korea (Registry to Evaluate the Efficacy of Zotarolimus-Eluting Stent) (NCT00960908) are multicenter prospective registries that consecutively enrolled 3,056 patients treated with everolimus-eluting stents (Xience V/Promus) from 29 centers (not sirolimus-eluting stents (Cypher)) and 1,998 patients treated with zotarolimus-eluting stents (Endeavor Resolute) from 25 centers, respectively, from 2008 through 2010. A total of 462 consecutive patients with LM bifurcation disease who underwent PCI using 2-stent strategies were identified: 181 were treated with the crush technique (22.2% classic, 63.2% mini-crush, and 14.6% other crush technique), 167 with T-stenting technique (14.4% classic T-stenting, 84.8% modified T or T and protrusion (TAP), and 0.8% inverted T-stenting), 32 with the culotte technique, and 81 with the kissing technique (Figure 1). This study complied with the provisions of the Declaration of Helsinki. The study protocol was approved by the institutional review board of each participating center. All patients provided written informed consent.

### 2.2. PCI Procedure

Coronary intervention was performed according to current standard procedural guidelines. The treatment strategy, details of the antiplatelet regimen, use of intravascular ultrasound, and choice of the specific DES type were left to the operator's discretion.

### 2.3. Definitions and Outcomes

The primary outcome was target lesion failure (TLF), a composite of cardiac death, myocardial infarction (MI), or target lesion revascularization (TLR). Secondary outcomes were patient-oriented composite outcome (POCO, a composite of all-cause death, any MI, stroke or any revascularization, individual elements of TLF and POCO, target vessel revascularization (TVR), and definite or probable stent thrombosis according to the Academic Research Consortium definitions.

**Figure 2:**
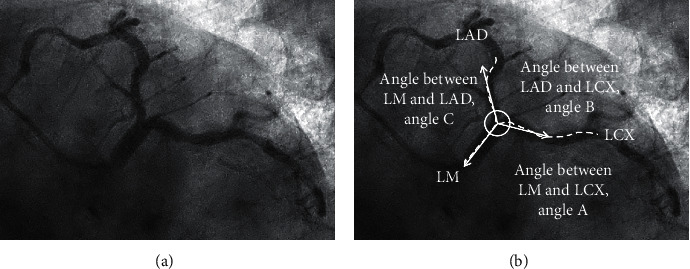
Measurement of left main bifurcation angles. Virtual lines were drawn from the branch origin under the LAO caudal view, and the 3 bifurcation angles were measured. LAD, left anterior descending artery; LAO, left anterior oblique; LCX, left circumflex artery; and LM, left main.

To reflect real-world practice, bifurcation angle was measured in an angiographic view with clear separation of bifurcation and least foreshortening, usually the left anterior oblique (LAO) caudal view. First, virtual lines were drawn as vectors extending from the branch origin. Next, the angles between LM and LAD (LM-LAD, angle C according to the European Bifurcation Club definition [18]), LAD and LCX (LAD-LCX, angle B), and LM and LCX (LM-LCX, angle A) were measured (Figure 2). The bifurcation angles were measured from preprocedural angiographic images. These bifurcation angles were independently assessed by 2 different cardiologists. The coefficient of variations (CVs) was calculated to determine the interobserver reliabilities for each bifurcation angle. Each CV was 20.0, 26.7, and 16.7, for the angle between LM and LCX, the angle between LAD and LCX, and the angle between LM and LAD, respectively, in our laboratory. The interobserver agreement for agreement on the wide angle between LM and LAD was 81.7% (Cohen's kappa = 0.553). The definition of procedural success was defined as a final residual stenosis <30% with TIMI flow grade 3 in either the main branch or the side branch.

### 2.4. Statistical Analysis

Data are expressed as numbers and percentages for categorical variables and as mean ± standard deviation (SD) for continuous variables. The differences in characteristics between groups were compared using chi-square tests for categorical variables and Student's *t*-test or one-way analysis of variance for continuous variables. Regarding categorical variables, the Fisher exact test was used when any expected cell count was less than 5 (not resulting from missing rows or columns in a larger table). To determine the best cutoff bifurcation angle to predict TLF, receiver operating characteristic (ROC) curve analysis was performed. In order to get the optimal cutoff values, we used the Youden index (= Sensitivity + Specificity − 1). The time-dependent event rate was estimated by the Kaplan–Meier method and compared with the log-rank test. If the combined end points occurred in one patient, the first event was counted. The Cox proportional hazard model was used to calculate the hazard ratios (HRs) for the endpoints. A multivariable Cox regression model was used to adjust for uneven distribution of baseline characteristics and to find independent predictors of the clinical outcome. Variables with *P* < 0.25 in the univariate analysis were included in multivariable Cox regression model. The final included variables are as follows: in the crush group, wide angle of LM-LAD (≥152°), MV calcification, long SB lesion (>5 mm), high SYNTAX score (≥33), final kissing ballooning (FKB), and true bifurcation And in the T-stenting group, current smoker, low LV systolic function (<50%), MV calcification, and long SB lesion (>5 mm). The final models were determined by the enter method. Results are reported as 95% confidence intervals (CIs). A two-sided value of *P* < 0.05 was considered significant for all probability values. SPSS version 22 (IBM SPSS Statistics, Chicago, IL, USA) was used for the statistical analyses.

## 3. Results

### 3.1. Baseline Clinical, Angiographic, and Procedural Characteristics

Of the 462 patients, 37% took the 2^nd^ generation DES, and the remainder, the 1^st^ generation DES. Because the majority of the study population underwent PCI using the crush technique or T-stenting, our study focused on these techniques. Baseline characteristics were similar between the crush technique and T-stenting group except for clinical manifestations: the acute coronary syndrome rate was higher in the crush group (crush versus T-stenting; 72.4% versus 54.5%; *P* < 0.001) (Table 1). Interestingly, although the choice between crush and T-stenting is generally determined based on LAD-LCX angle, the data from this real-world patient-level pooled registry showed no differences in bifurcation angles between the 2 groups (LM-LAD, 150.0 versus 155.2; *P*=0.061, LM-LCX, 121.1 versus 120.8; *P*=0.215, LAD-LCX, 82.0 versus 80.1; *P*=0.505, crush versus T-stenting, respectively).

**Figure 3:**
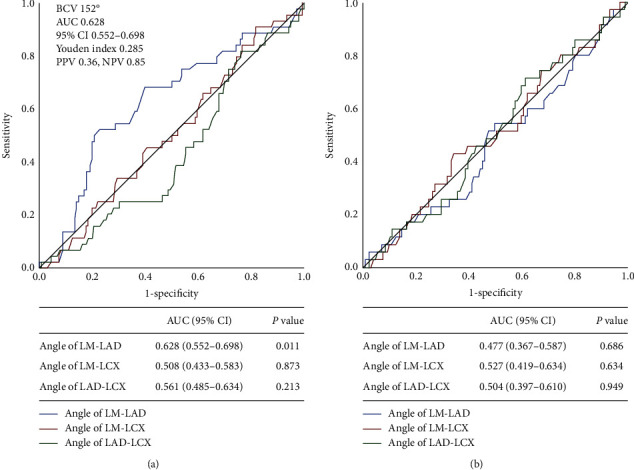
Receiver operating characteristic curves showing sensitivity of each angle for predicting TLF risk in patients treated with (a) crush and (b) T-stenting technique. An LM-LAD angle of 152° was the best cutoff value for predicting TLF in the crush group. None of the bifurcation angles had a significant cutoff value in the T-stenting group. AUC, area under the curve; BCV, best cutoff value; CI, confidence interval; LAD, left anterior descending artery; LCX, left circumflex artery; LM, left main; TLF, target lesion failure.

The median follow-up duration was 1,048 days (interquartile range (IQR), 641–1,578) for the whole population, 1,050 days (IQR, 671–1,598) for the crush group, and 1,095 days (IQR, 728–1,577) for the T-stenting group. ROC curve analysis revealed that an LM-LAD bifurcation angle of 152° is the best cutoff value to predict TLF in the crush group (area under the curve, 0.628; 95% CI, 0.552 to 0.698; *P*=0.011) (Figure 3). No significant cutoffs were found in other angles in the crush group. In contrast, no cutoff values were identified to predict TLF in any angles in the T-stenting group. Among patients receiving the crush technique, the prevalence of dyslipidemia was higher in the LM-LAD ≥152° group (Table 2). Among patients receiving T-stenting, the prevalence of hypertension and previous MI were higher in the LM-LAD ≥152° group than in the <152° group. Other clinical characteristics were not statistically different between the 2 groups in each technique. Angiographic and procedural characteristics were statistically similar between the 2 groups in each technique except for main vessel (MV) calcification in the crush technique and bifurcation angles in both (Table 3).

### 3.2. Clinical Outcomes Depending on Bifurcation Angles in Each Technique

**Figure 4:**
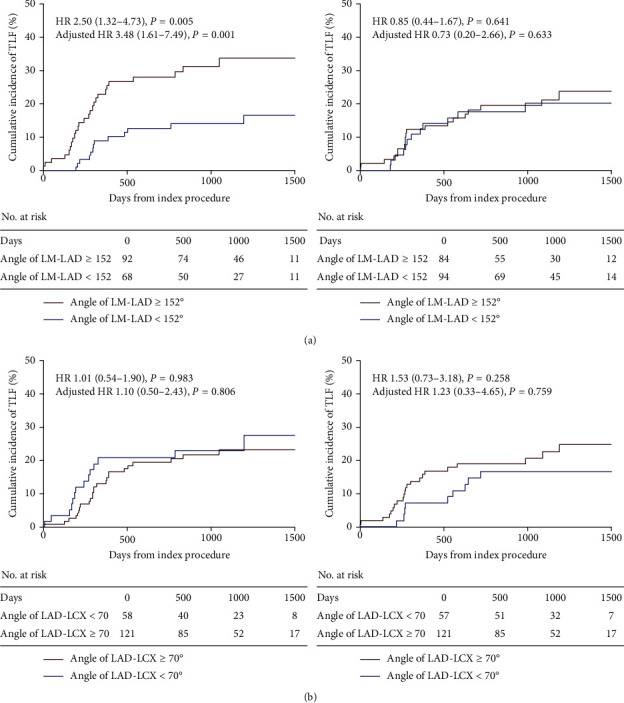
Clinical impact of bifurcation angle after LM PCI using the crush technique versus T-stenting. (a) The clinical impact of LM-LAD angle after left main percutaneous coronary intervention using the crush technique versus T-stenting. (b) The clinical impact of the LAD-LCX angle after left main percutaneous coronary intervention using the crush technique versus T-stenting. HR, hazard ratio; LAD, left anterior descending artery; LCX, left circumflex artery; LM, left main; and PCI, percutaneous coronary intervention. The left column represents the crush strategy, and the right column represents the T-stenting strategy.

TLF more frequently occurred in the LM-LAD angle ≥152° group than in the <152° group among patients treated with the crush technique (LM-LAD angle ≥152° versus <152°; 35.7% versus 14.6%, respectively; adjusted HR, 3.476; 95% CI, 1.612 to 7.492; *P*=0.001). In contrast, the incidence of TLF was not affected by an LM-LAD angle ≥152° or <152° in patients treated with the T-stenting technique (LM-LAD angle ≥152° versus <152°; 20.4% versus 22.5%; adjusted HR, 0.730; 95% CI, 0.200 to 2.663; *P*=0.633) (Figure 4(a), Table 4). The incidences of POCO, any revascularization, TLR, and TVR were significantly higher in the LM-LAD angle ≥152° group among patients treated with the crush technique. However, the incidences of other clinical outcomes were also similar between the 2 groups of patients treated with the T-stenting technique (Table 4). TLR occurred in 19.3% (*n* = 35) and 14.4% (*n* = 24) of the crush group and T-stenting group, respectively (*P*=0.213). Among the patients whose location of TLR sites was available, there were no statistical differences in TLR sites between the LM-LAD angle ≥152° and <152° group (Supplementary Table 1). When the crush technique was compared with T-stenting in the LM-LAD angle ≥152° and <152° group, respectively, the crush technique showed a higher tendency of TLF than T-stenting in the LM-LAD angle ≥152° group (Supplementary Figure 1).

In real-world practice, interventionists choose between T-stenting and crush techniques based on LAD-LCX angle, not LM-LAD. For bifurcation lesions with a LAD-LCX angle close to a right angle, the so-called wide LAD-LCX angle, T-stenting is preferred. In contrast, for a lesion with a narrow LAD-LCX angle, the crush technique is preferred. In this regard, the whole study population was reclassified by an LAD-LCX angle of 70°. However, the data showed that the incidence of TLF was not affected by an LAD-LCX angle ≥70° or <70° using either technique (Figure 4(b)).

Multivariable regression analysis revealed that an LM-LAD angle ≥152° was an independent predictor of TLF in the crush technique, whereas MV calcification was independent predictors of TLF in the T-stenting technique (Table 5). Neither the type of bifurcation (true or nontrue) nor generation of stent was an independent predictor of TLF in both techniques.

## 4. Discussion

To our knowledge, this is the first study to comprehensively evaluate the impact of 3 bifurcation angles in patients with LM bifurcation disease using the 2-stent strategy. The main findings of our study are as follows: (1) wide LM-LAD angle (≥152°) is associated with poor outcomes in LM bifurcation PCI using the crush technique and (2) outcomes of T-stenting in LM bifurcation lesion were not affected by any bifurcation angle.

There are several critical limitations in previous studies regarding angles of bifurcation PCI. First, bifurcation angle in other studies did not mean the same angle. Some studies referred to the angle between the MB and the SB (angle B) [16, 17, 19, 20], while others focused on the angle between the MV and the SB (angle A) [21]. Second, most dealt with non-LM bifurcation lesions [9, 16, 22, 23]. Third, some analyzed data from patients treated with the 1-stent strategy [24]. Fourth, all studies that suggested the bifurcation angle cutoff value for predicting poor outcomes arbitrarily selected the angles. Fifth, timing of measuring the angles (i.e., the systolic or diastolic phase, before or after PCI) was not standardized or specified in previous studies. As a result, previous studies showed mixed results as follows:

Dzavik et al. demonstrated that wide bifurcation angle (≥50°) was an independent predictor of major adverse cardiovascular events (MACE), a composite of death, MI, and TLR, in 133 patients treated with the crush technique [9]. In this study, only 6.0% of patients had LM disease. Furthermore, among them, it is unclear how many cases had LM disease as a main target for bifurcation PCI. A bifurcation angle of 50° was selected to stratify the study population because it was a median angle. This article is one of the earliest studies of angle in bifurcation PCI. Thereafter, it has been referred to in many articles as a study showing a negative impact of wide bifurcation angle between the MB and the SB (angle B). In fact, according to Section 2 of the article, this study defined the bifurcation angle as an angle between the MV and the SB (angle A). Thus, a bifurcation angle ≥50° in this study actually meant angle *A* < 130°. The same group also showed that a wide bifurcation angle (≥50°) was associated with a lower rate of MACE (death, MI, or TVR) for Canadian Cardiovascular Society (CCS) class ≥2 angina-free survival in patients treated with crush or Culotte stenting (*n* = 140) [10]. Only 5.7% of patients had LM disease as the main bifurcation target. Interestingly, outcomes of patients with MV stenting only (*n* = 266) were not affected by bifurcation angle. Again, although this study has been frequently misinterpreted as the one focusing on angle B (between the MB and the SB), it actually studied angle A (between the MV and the SB). Therefore, a bifurcation angle ≥50° in this study indicated an angle *A* < 130°. In the other study performed by the same group [11], the authors again demonstrated that a wide bifurcation angle (≥50°), indicating an angle *A* < 130°, was associated with a lower risk of MACE (death, MI, or TVR) or CCS class ≥2 angina in patients treated with crush or Culotte stenting (*n* = 360). Of the cohort, 3.1% of patients had LM disease as a main bifurcation target.

Adriaenssens et al. revealed that an increasing angle B (between the MB and the SB) was an independent predictor of angiographic restenosis in patients undergoing Culotte stenting (*n* = 134) [12]. This study excluded bifurcation interventions in the LM artery. Chen et al. analyzed 37 patients with unprotected LM bifurcation lesions treated with crush or double kissing (DK) crush stenting [13]. The data showed that an increasing bifurcation angle B (between the MB and the SB) was an independent predictor of TLR. The same group also demonstrated that an increasing bifurcation angle B was associated with a higher risk of MACE (cardiac death, MI, or TLR) in patients treated with crush stenting in another study (*n* = 230) [14]. This study included 33 cases (14.3%) of LM bifurcation interventions.

Interestingly, the same group compared the impact of a wide bifurcation angle B (≥60°) and narrow-angle B (<60°) in patients treated with crush or DK crush stenting (*n* = 220) in the other study and found that bifurcation angle B did not influence the clinical outcomes including MACE (cardiac death, MI, or TLR) [15]. A total of 15.2% of the study population underwent LM bifurcation intervention in this study. Yang et al. divided the patients undergoing bifurcation PCI into wide- and narrow-angle groups using median bifurcation angle B (50°) in their study (*n* = 1,432) [16]. The incidences of MACE (cardiac death, MI, or TLR) and TLR were not significantly different between the 2 groups. Although the study population was relatively large, this study focused on only non-LM bifurcation lesions. Furthermore, the vast majority of patients (84.5%) were treated with the 1-stent technique. Girasis et al. stratified the patients receiving LM bifurcation PCI by tertiles of angle B (<82°, 82–106°, ≥107°) [17]. The results showed that angle B did not affect the rate of major adverse cardiac and cerebrovascular events (a composite of all-cause death, cerebrovascular accident, MI, or repeat revascularization) in patients treated with 1 stent (*n* = 75) and those treated with ≥2 stents (*n* = 110).

In summary, previous studies reported mixed results with mixed definitions of bifurcation angle.

The current study comprehensively analyzed the impact of each of 3 bifurcation angles on clinical outcomes in patients receiving LM bifurcation PCI with DES using the major 2-stent techniques (crush and T-stenting). Our data revealed that a wide LM-LAD (angle C) was an independent predictor of worse outcomes in the crush technique, whereas bifurcation angle did not affect the T-stenting outcomes. The cutoff value of an LM-LAD of 152° for predicting TLF was statistically determined using ROC curve analysis.

The current practical guides recommend a provisional approach with a simple crossover technique using a 1-stent rather than an upfront 2-stent technique [3, 25]. However, considering the diameter of the LCX artery, the area it supplies, and the significance of its flow preservation, an LM bifurcation lesion is the bifurcation where the need for use of the 2-stent strategy is underscored compared to other bifurcations. Our study provides useful information for LM bifurcation PCI using the 2-stent technique.

**Figure 5:**
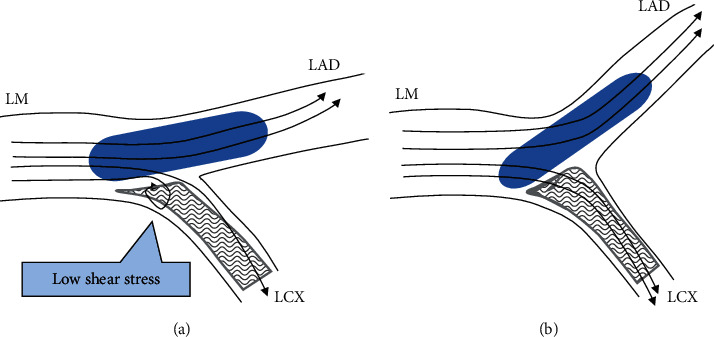
Schematic diagrams of LM bifurcation according to different LM-LAD angles. Blue ovals mean the balloon catheter, gray rectangles mean the stent, and arrows indicate wall shear stress. (a) Wide LM-LAD angle. (b) Narrow LM-LAD angle. LAD, left anterior descending artery; LCX, left circumflex artery; and LM, left main.

The reason why a wide LM-LAD angle (≥152°) is associated with poor crush technique outcomes is unclear. The first possible explanation for our findings is the potential uneven expansion of kissing balloons. In bifurcation lesions with a wide LM-LAD angle, a kissing balloon in LM-LAD can be easily straightened, whereas a bending force may be placed on the opposite side of a balloon in LM-LCX, resulting in uneven expansion. This effect may be prominent in the crush technique, in which the optimization of crushed struts may be more important than in T-stenting. Although FKB was not a predictor of events (Table 5) in the crush group, it may be because kissing ballooning could not be adequately performed in patients taking FKB. Second, crush of the side branch stent in the bifurcation with a wide LM-LAD angle could be incomplete due to weak force of the straightened balloon (Figure 5(a)). Third, in the bifurcation with a wide LM-LAD angle, relatively lower shear stress area could be generated in the lateral side of LM where the crushed stent struts are located (Figure 5(a)). In contrast, in the bifurcation with a narrow LM-LAD angle, shear stress could be ideally distributed (Figure 5(b)). All these potential mechanisms are hypotheses from scientific speculation which need validation with further studies.

## 5. Limitations

The current study has some limitations. First, because this study was based on registry data, there are intrinsic limitations of nonrandomized comparisons including biased distribution of risk factors and lesion characteristics and possible influences of unmeasured confounding factors despite multivariable adjustment. Second, the selection of stenting techniques was left entirely to the operator's discretion, reflecting the registry nature of our study. This may cause selection bias, although clinical, angiographic, and procedural characteristics were fairly evenly distributed across the groups (Tables 2 and 3). In addition, the outcomes of LM bifurcation PCI could be dependent on the expertise of the operator. However, this variable was not available in our pooled registry. Third, we used 2-dimensional QCA to measure bifurcation angles. Three-dimensional QCA was recently suggested as a useful tool for the accurate and precise measurement of bifurcation angles. However, we think that 2-dimensional QCA may better reflect the results of daily practice since visual estimation remains the most frequently and widely adopted method in the real world. Fourth, this study was conducted with relatively small numbers of study population. However, to our knowledge, this is the largest study investing the impact of bifurcation angles in LM bifurcation treated with the two-stent technique. Fifth, about two-thirds of the study population in our study received the 1^st^ generation DES. Although multivariable regression analysis showed the generation of DES was not an independent predictor of TLF (Table 5), high proportion of the 1^st^ generation DES may not reflect good outcomes of the current practice. Lastly, the underlying mechanisms for poor outcomes in a wide LM-LAD angle were limited. Further studies using *in vitro* or *in silico* models are warranted.

## 6. Conclusions

In patients undergoing LM bifurcation PCI using the 2-stent technique with DES, a wide LM-LAD angle (≥152°) was associated with a greater risk of TLF in patients treated with the crush technique, mainly driven by an increased TLR rate, whereas none of the bifurcation angles affected T-stenting outcomes.

## Figures and Tables

**Table 1:** Baseline clinical, angiographic, and procedural characteristics of the study population.

Clinical characteristics	Total (*n* = 348)	Crush (*n* = 181)	T-stenting (*n* = 167)	*P* value
Age, years	64.4 ± 10.0	64.4 ± 9.9	64.5 ± 10.2	0.944
Male	207 (59.5)	110 (60.8)	97 (58.1)	0.610
Diabetes mellitus	112 (32.2)	59 (32.6)	53 (31.7)	0.864
Hypertension	203 (58.3)	107 (59.1)	96 (57.5)	0.758
Dyslipidemia	105 (30.2)	47 (26.0)	58 (34.7)	0.075
Peripheral vascular disease	9 (2.6)	5 (2.8)	4 (2.4)	1.000
Chronic kidney disease^*∗*^	30 (8.6)	19 (10.5)	11 (6.6)	0.194
Current smoker	74 (21.3)	42 (23.2)	32 (19.2)	0.357
Previous myocardial infarction	27 (7.8)	16 (8.8)	11 (6.6)	0.433
Previous cerebrovascular event	29 (8.3)	18 (9.9)	11 (6.6)	0.258
Previous PCI	79 (22.7)	47 (26.0)	32 (19.2)	0.130
Previous CABG	6 (1.7)	4 (2.2)	2 (1.2)	0.686
Family history of CAD	15 (4.3)	8 (4.4)	7 (4.2)	0.917
LV ejection fraction, %	58.2 ± 11.8	58.7 ± 12.2	57.5 ± 11.2	0.411
*Clinical Manifestation*				0.005
STEMI	22 (6.3)	11 (6.1)	11 (6.6)	
NSTEMI	35 (10.1)	26 (14.4)	9 (5.4)	
Unstable angina	165 (47.4)	94 (51.9)	71 (42.5)	
Stable angina	117 (33.6)	47 (26.0)	70 (41.9)	
Silent ischemia	8 (2.3)	3 (1.7)	5 (3.0)	

*Angiographic and Procedural Characteristics*				
Angle of LM-LAD	152.4 (135.0–166.0)	150.0 (134.0–165.9)	155.2 (138.0–166.3)	0.061
Angle of LM-LCX	121.0 (105.4–137.0)	121.1 (106.6–139.1)	120.8 (105.0–136.2)	0.215
Angle of LAD-LCX	81.0 (64.0–102.0)	82.0 (64.0–105.0)	80.1 (64.2–101.0)	0.505
*SYNTAX Score*				0.019
Low score (0–22)	120 (34.7)	59 (32.6)	61 (37.0)	
Intermediate score (23–32)	151 (43.6)	72 (39.8)	79 (47.9)	
High score (≥33)	75 (21.7)	50 (39.8)	25 (15.2)	
*Medina classification*				0.084
*True Bifurcation*				
1.1.1	161 (46.4)	76 (42.0)	85 (50.9)	
1.0.1	33 (9.5)	21 (11.6)	12 (7.2)	
0.1.1	66 (14.3)	22 (12.2)	27 (16.2)	
*Nontrue Bifurcation*				
1.0.0	9 (2.6)	4 (2.2)	5 (3.0)	
0.1.0	26 (7.5)	16 (8.8)	10 (6.0)	
1.1.0	38 (11.0)	17 (9.4)	21 (12.7)	
0.0.1	31 (9.3)	25 (13.8)	6 (3.6)	
*DES Type*				0.146
SES	151 (43.4)	81 (44.8)	70 (41.9)	
PES	63 (18.1)	33 (18.2)	30 (18.0)	
ZES	58 (16.7)	26 (14.4)	32 (19.2)	
EES	56 (16.1)	34 (18.8)	22 (13.2)	
BP-BES	18 (5.2)	6 (3.3)	12 (7.2)	
*Stent Type*				0.636
First generation DES	217 (62.4)	115 (63.5)	102 (61.1)	
Second generation DES	131 (37.6)	66 (36.5)	65 (38.9)	
LAD stent diameter	3.4 ± 0.4	3.4 ± 0.4	3.4 ± 0.4	0.167
LCX stent diameter	3.0 ± 0.4	3.0 ± 0.4	3.1 ± 0.4	0.047

Values are mean ± SD, median (interquartile ranges, 25^th^–75^th^), or *n* (%) (per-patient analysis). ^*∗*^Chronic kidney disease defined as a glomerular filtration rate (GFR) < 60 ml/min/1.73 m^2^. BP-BES, biodegradable polymer biolimus-eluting stent; CABG, coronary artery bypass grafting; CAD, coronary artery disease; DES, drug-eluting stent; EES, everolimus-eluting stent; LAD, left anterior descending artery; LCX, left circumflex artery; LM, left main; LV, left ventricle; NSTEMI, non-ST-segment elevation myocardial infarction; PCI, percutaneous coronary intervention; PES, paclitaxel-eluting stent; SES, sirolimus-eluting stent; STEMI, ST-segment elevation myocardial infarction; ZES, zotarolimus-eluting stent.

**Table 2:** Baseline clinical characteristics in patients treated with the 2-stent technique using the crush technique or T-stenting in LM bifurcation.

	Crush (*n* = 181)			T-stenting (*n* = 167)		
	LM-LAD angle ≥152° (*n* = 84)	LM-LAD angle <152° (*n* = 96)	*P* value	LM-LAD angle ≥152° (*n* = 93)	LM-LAD angle <152° (*n* = 71)	*P* value
Age, years	63.1 ± 11.1	65.4 ± 8.6	0.124	65.0 ± 9.0	64.0 ± 11.5	0.535
Male	55 (65.5)	54 (56.3)	0.224	57 (61.3)	37 (52.1)	0.239
Diabetes mellitus	24 (28.6)	35 (36.5)	0.271	33 (35.5)	18 (25.4)	0.165
Hypertension	48 (57.1)	58 (60.4)	0.384	60 (64.5)	35 (49.3)	0.050
Dyslipidemia	28 (33.3)	19 (19.8)	0.029	30 (32.3)	27 (38.0)	0.442
Peripheral vascular disease	2 (2.4)	3 (3.1)	1.000	4 (4.3)	0 (0)	0.134
Chronic kidney disease^*∗*^	7 (8.3)	12 (12.5)	0.468	6 (6.5)	5 (7.0)	1.000
Current smoker	23 (27.4)	19 (19.8)	0.289	22 (23.7)	9 (12.7)	0.075
Previous myocardial infarction	5 (6.0)	11 (11.5)	0.294	10 (10.8)	1 (1.4)	0.024
Previous cerebrovascular event	10 (11.9)	8 (8.3)	0.464	6 (6.5)	4 (5.6)	1.000
Previous PCI	20 (23.8)	27 (28.1)	0.610	22 (23.7)	10 (14.1)	0.125
Previous CABG	2 (2.4)	2 (2.1)	1.000	2 (2.2)	0 (0)	0.506
Family history of CAD	4 (4.8)	4 (4.2)	1.000	4 (4.3)	2 (2.8)	0.699
LV ejection fraction, %	59.8 ± 12.4	57.8 ± 12.1	0.310	57.9 ± 9.7	57.9 ± 12.6	0.985
*Clinical Manifestation*			0.359			0.645
STEMI	7 (8.3)	4 (4.2)		7 (7.5)	4 (5.6)	
NSTEMI	13 (15.5)	13 (13.5)		6 (6.5)	2 (2.8)	
Unstable angina	41 (48.8)	41 (54.2)		41 (44.1)	30 (42.3)	
Stable angina	23 (27.4)	27 (28.1)		39 (42.0)	31 (43.7)	

Values are mean ± standard deviations, median (interquartile ranges, 25^th^–75^th^), or *n* (%) (per-patient analysis). ^*∗*^Chronic kidney disease defined as a glomerular filtration rate (GFR) < 60 ml/min/1.73 m^2^. CABG, coronary artery bypass grafting; CAD, coronary artery disease; LAD, left anterior descending artery; LM, left main; LV, left ventricle; NSTEMI, non-ST-segment elevation myocardial infarction; PCI, percutaneous coronary intervention; STEMI, ST-segment elevation myocardial infarction.

**Table 3:** Angiographic and procedural characteristics in patients treated with the 2-stent technique using the crush technique or T-stenting in LM Bifurcation.

	Crush			T-stenting		
	LM-LAD angle ≥152° (*n* = 84)	LM-LAD angle <152° (*n* = 96)	*P* value	LM-LAD angle ≥152° (*n* = 93)	LM-LAD angle <152° (*n* = 71)	*P* value
Angle of LM-LAD	166.4 (158.5–175.1)	134.0 (120.5–143.2)	<0.001	165.0 (157.6–173.3)	136.1 (125.0–142.0)	<0.001
Angle of LM-LCX	116.2 (104.1–131.8)	125.5 (108.7–143.0)	0.009	120.1 (104.0–132.7)	121.0 (107.0–137.0)	0.495
Angle of LAD-LCX	68.3 (55.8–86.7)	94.5 (79.5–116.5)	<0.001	71.9 (60.4–85.1)	96.0 (76.1–109.8)	<0.001
*SYNTAX Score*			0.972			0.690
Low score (0–22)	27 (32.1)	31 (32.2)		32 (34.8)	28 (40.0)	
Intermediate score (23–32)	33 (39.3)	39 (40.6)		47 (51.1)	31 (44.3)	
High score (≥33)	24 (28.6)	26 (27.1)		13 (14.1)	11 (15.7)	
*Medina Classification*			0.603			0.475
True bifurcation						
1.1.1	38 (45.2)	38 (39.6)		42 (45.2)	42 (59.2)	
1.0.1	10 (11.9)	11 (11.5)		7 (7.5)	5 (7.0)	
0.1.1	9 (10.7)	13 (13.5)		18 (19.4)	9 (12.7)	
Nontrue bifurcation						
1.0.0	2 (2.4)	2 (2.1)		2 (2.2)	2 (2.8)	
0.1.0	6 (7.1)	10 (10.4)		7 (7.5)	3 (4.2)	
1.1.0	5 (6.0)	12 (12.5)		12 (12.9)	8 (11.3)	
0.0.1	14 (16.7)	10 (10.4)		5 (5.4)	1 (1.4)	
*DES Type*			0.609			0.775
SES	40 (47.6)	41 (42.7)		43 (46.2)	27 (38.0)	
PES	18 (21.4)	15 (15.6)		15 (16.1)	15 (21.1)	
ZES	9 (10.7)	16 (16.7)		13 (14.3)	9 (12.7)	
EES	15 (17.9)	19 (19.8)		15 (16.1)	14 (19.7)	
BP-BES	0 (0)	1 (1.0)		6 (6.5)	6 (8.5)	
*Stent Type*			0.267			0.442
First generation DES	60 (71.4)	60 (62.5)		63 (67.7)	44 (62.0)	
Second generation DES	24 (28.6)	36 (37.5)		30 (32.3)	27 (38.0)	
LAD stent diameter	3.4 ± 0.4	3.5 ± 0.4	0.408	3.4 ± 0.4	3.4 ± 0.4	0.655
LCX stent diameter	3.0 ± 0.3	3.0 ± 0.3	0.990	3.2 ± 0.5	3.2 ± 0.4	0.410
IVUS-guided PCI	53 (63.1)	53 (55.2)	0.293	66 (71.0)	49 (69.0)	0.787
Rotablation	0 (0)	1 (1.0)	1.000	1 (1.1)	1 (1.4)	1.000
Final kissing ballooning	65 (83.3)	73 (83.0)	0.948	73 (90.1)	60 (96.8)	0.187
Conversion from provisional stenting	0/76 (0)	3/75 (4.0)	0.120	60/79 (75.9)	47/53 (88.7)	0.067
Main vessel procedural success	84/84 (100.0)	95/96 (99.0)	1.000	93/93 (100.0)	70/70 (100.0)	NA
Side branch procedural success	84/84 (100.0)	95/96 (99.0)	1.000	93/93 (100.0)	68/70 (97.1)	0.183
*Preintervention QCA*						
MV RD, mm	3.2 ± 0.4	3.2 ± 0.5	0.602	3.3 ± 0.5	3.2 ± 0.6	0.718
SB RD, mm	2.6 ± 0.4	2.7 ± 0.4	0.262	2.9 ± 0.5	2.7 ± 0.6	0.114
MV MLD, mm	1.2 ± 0.6	1.2 ± 0.6	0.719	1.2 ± 0.6	1.1 ± 0.5	0.324
SB MLD, mm	1.2 ± 0.6	1.1 ± 0.6	0.572	1.3 ± 0.6	1.1 ± 0.5	0.055
MV diameter stenosis, %	62.1 ± 17.2	64.4 ± 16.0	0.359	62.2 ± 18.1	64.8 ± 17.8	0.354
SB diameter stenosis, %	56.2 ± 20.5	59.4 ± 20.5	0.306	54.1 ± 19.4	58.5 ± 18.4	0.161
MV lesion length, mm	21.5 ± 17.5	25.4 ± 20.3	0.175	20.4 ± 14.0	20.1 ± 14.9	0.911
SB lesion length, mm	12.8 ± 11.4	12.6 ± 11.8	0.919	9.9 ± 10.4	11.9 ± 12.3	0.260
MV calcification	22 (26.2)	42 (43.8)	0.019	36 (38.7)	28 (40.0)	0.867
SB calcification	15 (17.9)	21 (21.9)	0.577	21 (22.6)	14 (20.0)	0.671
*Postintervention QCA*						
MV RD, mm	3.2 ± 0.5	3.2 ± 0.6	0.806	3.5 ± 0.6	3.3 ± 0.6	0.167
SB RD, mm	2.7 ± 0.5	2.7 ± 0.5	0.753	2.9 ± 0.5	2.8 ± 0.5	0.214
MV MLD, mm	2.8 ± 0.5	2.7 ± 0.5	0.492	2.7 ± 0.5	2.9 ± 0.6	0.521
SB MLD, mm	2.5 ± 0.5	2.5 ± 0.5	0.416	2.7 ± 0.6	2.6 ± 0.5	0.292
MV diameter stenosis, %	14.6 ± 11.5	16.3 ± 11.8	0.387	15.3 ± 11.4	14.7 ± 9.7	0.765
SB diameter stenosis, %	6.2 ± 17.8	7.9 ± 14.7	0.537	9.1 ± 13.8	8.3 ± 12.6	0.735

Values are mean ± standard deviations, median (interquartile ranges, 25^th^–75^th^), or *n* (%) (per-patient analysis). BP-BES, biodegradable polymer biolimus-eluting stent; DES, drug-eluting stent; EES, everolimus-eluting stent; IVUS, intravascular ultrasound; LAD, left anterior descending artery; LCX, left circumflex artery; LM, left main; MLD, minimal lumen diameter; MV, main vessel; PCI, percutaneous coronary intervention; PES, paclitaxel-eluting stent; QCA, quantitative coronary angiography; RD, reference diameter; SB, side branch; SES, sirolimus-eluting stent; ZES, zotarolimus-eluting stent.

**Table 4:** Adjusted hazard ratios of wide (≥152°) compared with narrow LM-LAD angle (<152°) in patients treated with the crush technique and with T-stenting.

*Crush strategy*	LM-LAD angle ≥152°	LM-LAD angle <152°	Adjusted HR (95% CI)	*P* value
Target lesion failure^*∗*^	30 (35.7)	14 (14.6)	3.476 (1.612–7.492)	0.001
Patient-oriented composite outcome^†^	38 (45.2)	27 (28.1)	2.061 (1.126–3.772)	0.019
All-cause death	7 (8.3)	11 (11.5)	0.517 (0.126–2.118)	0.360
Cardiac death	5 (6.0)	3 (3.1)	4.661 (0.182–119.518)	0.352
Spontaneous MI	3 (3.6)	1 (1.0)	5.506 (0.118–257.567)	0.385
Any revascularization	31 (36.9)	17 (17.7)	2.849 (1.379–5.889)	0.005
Target lesion revascularization	24 (28.6)	11 (11.5)	3.758 (1.602–8.817)	0.002
Target vessel revascularization	30 (35.7)	14 (14.6)	2.404 (1.117–5.176)	0.025
Definite or probable stent thrombosis	4 (4.8)	2 (2.1)	1.885 (0.203–17.534)	0.577

*T-Stent Strategy*				
Target lesion failure^*∗*^	19 (20.4)	16 (22.5)	0.730 (0.200–2.663)	0.633
Patient-oriented composite outcome^†^	26 (28.0)	27 (38.0)	0.745 (0.291–1.907)	0.539
All-cause death	12 (12.9)	9 (12.7)	0.714 (0.152–3.351)	0.669
Cardiac death	6 (6.5)	4 (5.6)	0.501 (0.024–10.478)	0.656
Spontaneous MI	3 (3.2)	2 (2.8)	0 (0-indefinite)	0.881
Any revascularization	17 (18.3)	17 (23.9)	1.326 (0.393–4.466)	0.649
Target lesion revascularization	14 (15.1)	10 (14.1)	0.660 (0.118–3.686)	0.636
Target vessel revascularization	17 (18.3)	15 (21.1)	1.578 (0.383–6.498)	0.527
Definite or probable stent thrombosis	4 (4.3)	2 (2.8)	0.182 (0.002–18.727)	0.471

^*∗*^Target lesion failure defined as a composite of cardiac death, myocardial infarction, or target lesion revascularization. ^†^Patient-oriented composite outcomes defined as a composite of cardiac death, myocardial infarction, stroke, or any revascularization. Variables included in the Cox proportional hazard regression model were wide LM-LAD angle (≥152°), wide LAD-LCX angle (≥70°), diabetes mellitus, dyslipidemia, current smoker, low LV systolic function (<50%), chronic kidney disease, acute coronary syndrome, main vessel calcification, long side branch lesion (>5 mm), high SYNTAX score (≥33), and final kissing ballooning. CI, confidence interval; HR, hazard ratio; LAD, left anterior descending artery; LCX, left circumflex artery; LM, left main; LV, left ventricle; MI, myocardial infarction.

**Table 5:** Independent predictors of TLF in patients treated with the crush technique and T-stenting.

	Univariable analysis		Multivariable analysis	
	HR (95% CI)	*P* value	HR (95% CI)	*P* value
*Crush Technique*				
Wide angle of LM-LAD (≥152°)	2.50 (1.32–4.73)	0.005	2.57 (1.34–4.90)	0.004
Wide angle of LAD-LCX (≥70°)	1.01 (0.54–1.90)	0.983	—	—
Diabetes mellitus	1.18 (0.63–2.19)	0.613	—	—
Dyslipidemia	1.27 (0.67–2.40)	0.461	—	—
Current smoker	0.96 (0.46–1.99)	0.907	—	—
Low LV systolic function (<50%)	1.27 (0.62–2.58)	0.509	—	—
Chronic kidney disease	1.36 (0.53–3.47)	0.521	—	—
Acute coronary syndrome	1.20 (0.63–2.28)	0.577	—	—
MV calcification	1.55 (0.85–2.81)	0.152	1.60 (0.86–3.00)	0.138
Long SB lesion (>5 mm)	1.98 (0.92–4.26)	0.081	1.57 (0.70–3.51)	0.275
High SYNTAX score (≥33)	2.52 (1.16–5.46)	0.019	1.80 (0.97–3.33)	0.062
Final kissing ballooning	0.97 (0.41–2.34)	0.953	—	—
IVUS-guided PCI	0.95 (0.52–1.73)	0.874	—	—
True bifurcation	1.63 (0.82–3.22)	0.162	1.28 (0.63–2.61)	0.501
2^nd^ generation DES	1.42 (0.73–2.76)	0.308	—	—

*T-stenting technique*				
Wide angle of LM-LAD (≥152°)	0.85 (0.44–1.67)	0.641	—	—
Wide angle of LAD-LCX (≥70°)	1.53 (0.73–3.18)	0.258	—	—
Diabetes mellitus	0.97 (0.46–2.01)	0.926	—	—
Dyslipidemia	1.17 (0.59–2.33)	0.656	—	—
Current smoker	2.17 (1.04–4.52)	0.039	1.94 (0.82–4.58)	0.129
Low LV systolic function (<50%)	2.33 (1.01–5.37)	0.047	2.08 (0.86–5.00)	0.103
Chronic kidney disease	1.50 (0.46–4.91)	0.506	—	—
Acute coronary syndrome	0.83 (0.42–1.61)	0.578	—	—
MV calcification	2.31 (1.18–4.52)	0.014	2.78 (1.20–6.41)	0.017
Long SB lesion (>5 mm)	1.81 (0.85–3.87)	0.125	1.12 (0.47–2.70)	0.796
High SYNTAX score (≥33)	1.68 (0.34–3.41)	0.912	—	—
Final kissing ballooning	0.75 (0.18–3.17)	0.694	—	—
IVUS-guided PCI	0.94 (0.45–1.96)	0.859	—	—
True bifurcation	1.47 (0.64–3.36)	0.365	—	—
2^nd^ generation DES	0.87 (0.44–1.72)	0.697	—	—

CI, confidence interval; DES, drug-eluting stent; HR, hazard ratio; LAD, left anterior descending artery; LCX, left circumflex artery; LM, left main; LV, left ventricle; MV, main vessel; SB, side branch; TLF, target lesion failure.

## Data Availability

The clinical and procedural data used to support the findings of this study are included within the article.

## Supplementary Materials

Supplementary Materials
*Supplementary Table 1*. Target lesion revascularization sites in each left main bifurcation group. *Supplementary Figure 1*. TLF according to the stent techniques in patients with wide (≥152°) and narrow LM-LAD angle (<152°).
