# Supplementary material for: Clinical Implications of Bifurcation Angles in Left Main Bifurcation Intervention Using a Two-Stent Technique
Source: J Interv Cardiol. 2020 Jul 11;2020:2475930. doi: 10.1155/2020/2475930 (PMC7374237; doi:10.1155/2020/2475930)
Supplement: Supplementary Materials — Supplementary Table 1. Target lesion revascularization sites in each left main bifurcation group. Supplementary Figure 1. TLF according to the stent techniques in patients with wide (≥152°) and narrow LM-LAD angle (<152°). [file 2475930.f1.docx]

**Supplement to:**

**Clinical implications of bifurcation angles in left main bifurcation intervention using a two-stent technique**

You-Jeong Ki, MD, Ji Hyun Jung, MD, Jung-Kyu Han, MD, PhD, Sukkeun Hong, MD, PhD, Jang Hyun Cho, MD,

Hyeon-Cheol Gwon, MD, PhD, Sung Yun Lee, MD, PhD, Rhew Jay Young, MD, PhD, Jei Keon Chae, MD, PhD, In-Ho Chae, MD, PhD,

Han-Mo Yang, MD, PhD, Kyung Woo Park, MD, PhD, Hyun-Jae Kang, MD, PhD, Bon-Kwon Koo, MD, PhD, Hyo-Soo Kim, MD, PhD.

**Contents:**

Page 2: Supplementary Table 1. Target lesion revascularization sites in each left main bifurcation group

Page 3: Supplementary Figure 1. TLF according to the stent techniques in Patients with Wide (≥152°) and Narrow LM-LAD angle (<152°)

**Supplementary Table 1. Target lesion revascularization sites in each left main bifurcation group**

|  | **Crush (n=25)** | | |  | **T-stenting (n=16)** | | |
| --- | --- | --- | --- | --- | --- | --- | --- |
|  | **Angle LM to LAD ≥ 152**  **(n=18)** | **Angle LM to LAD < 152**  **(n=7)** | ***P* value** |  | **Angle LM to LAD ≥ 152**  **(n=9)** | **Angle LM to LAD < 152**  **(n=7)** | ***P* value** |
|  |  |  | 0.613 |  |  |  | 0.530 |
| LAD | 1 (5.6%) | 1 (14.3%) |  |  | 0 (0%) | 1 (14.3%) |  |
| LCX | 8 (44.4%) | 4 (57.1%) |  |  | 6 (66.7%) | 5 (71.4%) |  |
| Both LAD and LCX | 4 (22.2%) | 2 (28.6%) |  |  | 2 (22.2%) | 1 (14.3%) |  |
| LM | 3 (16.7%) | 0 (0%) |  |  | 0 (0%) | 0 (0%) |  |
| LM and LAD | 1 (5.6%) | 0 (0%) |  |  | 0 (0%) | 0 (0%) |  |
| LM, LAD and LCX | 1 (5.6%) | 0 (0%) |  |  | 1 (11.1%) | 0 (0%) |  |

LAD, left anterior descending artery; LCX, left circumflex artery; LM, left main.

**Supplementary Figure 1. TLF according to the stent techniques in Patients with Wide (≥152°) and Narrow LM-LAD angle (<152°)**


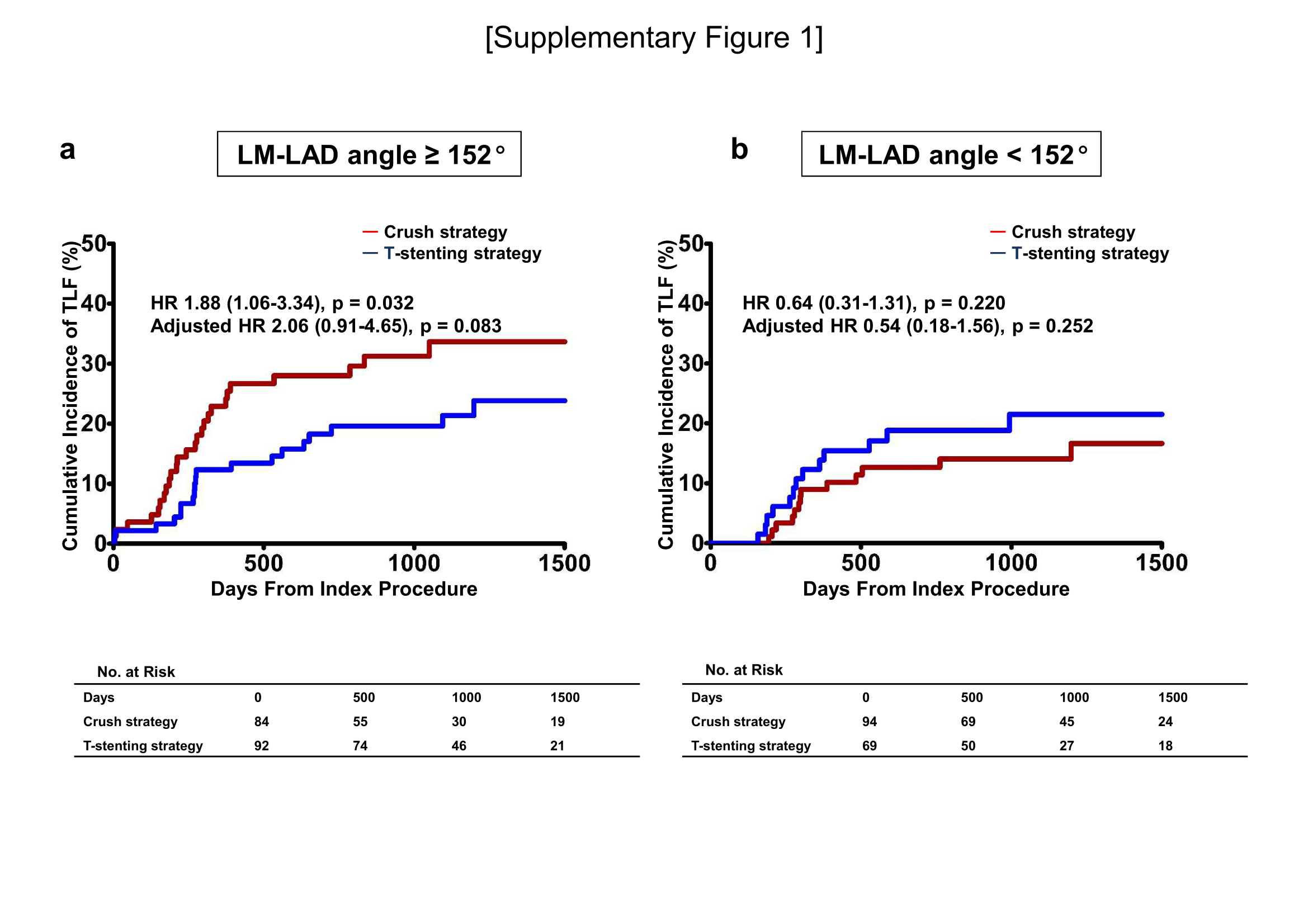


Variables included in Cox proportional hazard regression model were PCI technique, diabetes mellitus, dyslipidemia, current smoker, low LV systolic function (<50%), chronic kidney disease, acute coronary syndrome, main vessel calcification, long side branch lesion (>5mm), high SYNTAX score (≥33), and final kissing ballooning.

HR, hazard ratio; LAD, left anterior descending artery; LCX, left circumflex artery; LM, left main; TLF, target lesion failure.
